# Nanoparticle delivery to metastatic breast cancer cells by nanoengineered mesenchymal stem cells

**DOI:** 10.3762/bjnano.9.32

**Published:** 2018-01-29

**Authors:** Liga Saulite, Karlis Pleiko, Ineta Popena, Dominyka Dapkute, Ricardas Rotomskis, Una Riekstina

**Affiliations:** 1 Faculty of Medicine, University of Latvia, Raina Blvd. 19, LV-1586 Riga, Latvia; 2 Biomedical Physics Laboratory, National Cancer Institute, P. Baublio Street 3b, LT-08406 Vilnius, Lithuania; 3 Life Science Center, Vilnius University, Sauletekio Ave. 7, LT-10257 Vilnius, Lithuania,; 4 Laser Research Centre, Vilnius University, Sauletekio al. 9, corp. 3, LT-10222 Vilnius, Lithuania

**Keywords:** cancer, mesenchymal stem cells, quantum dots, spheroids, 3D cell culture

## Abstract

We created a 3D cell co-culture model by combining nanoengineered mesenchymal stem cells (MSCs) with the metastatic breast cancer cell line MDA-MD-231 and primary breast cancer cell line MCF7 to explore the transfer of quantum dots (QDs) to cancer cells. First, the optimal conditions for high-content QD loading in MSCs were established. Then, QD uptake in breast cancer cells was assessed after 24 h in a 3D co-culture with nanoengineered MSCs. We found that incubation of MSCs with QDs in a serum-free medium provided the best accumulation results. It was found that 24 h post-labelling QDs were eliminated from MSCs. Our results demonstrate that breast cancer cells efficiently uptake QDs that are released from nanoengineered MSCs in a 3D co-culture. Moreover, the uptake is considerably enhanced in metastatic MDA-MB-231 cells compared with MCF7 primary breast cancer cells. Our findings suggest that nanoengineered MSCs could serve as a vehicle for targeted drug delivery to metastatic cancer.

## Introduction

The recent progress in the development of nanoscale agents opens up new perspectives for targeted drug delivery in cancer diagnostics, imaging and therapy. However, once administered into the body, nanoparticles (NPs) are rapidly phagocytosed by the macrophages and sequestered in the liver, spleen, and lymph nodes [1–2]. To overcome this hurdle, targeted drug delivery using nanoengineered cells with cancer homing capability has emerged as an alternative approach. Based on the characteristic tumour tropism, integration in tumour stroma and their immune privileged nature, mesenchymal stem cells (MSCs) can be used as a delivery vehicle for therapeutic and imaging agents, such as drug-conjugated NPs [3–4]. MSCs are adult stem cells that can be isolated from various organs, including brain, liver, kidney, lung, bone marrow, muscle, thymus, skin, adipose tissue, umbilical cord and placenta [5]. MSCs express CD105 (SH2 or endoglin), CD73 (SH3 and SH4), CD106 (VCAM-1), CD44 (hyaluronic acid receptor), CD90 (Thy 1.1), CD29, CD146 and CD166 surface markers and can be induced to differentiate in vitro into diverse lineages of mesodermal origin, such as adipogenic, osteogenic and chondrogenic cells [6].

MSC-based therapies are being increasingly investigated for their promising potential in cancer diagnostics and treatment [5,7–8]. In vivo studies have demonstrated that MSCs can effectively deliver nanorattle-encapsulated doxorubicin to U251 glioma cells and induce cancer cell apoptosis [9]. Moreover, MSCs carrying poly(lactic-*co*-glycolic acid) (PLGA) NPs linked with paclitaxel selectively accumulate in an orthotopic A549 lung tumour model [2]. It has been reported that IFN-beta secreting MSCs could integrate into A375SM melanoma tumours to inhibit the growth of cancer cells [10]. Bioluminescent imaging has demonstrated the MSC tumour homing ability in an in vivo xenogeneic breast carcinoma and ovarian tumour model [11]. Lourenco et al. demonstrated that the tumour homing capability in MSCs is induced by MIF–CXCR4 chemotaxis and downstream activation of the MAPK pathway [12]. QD-loaded MSCs have shown to migrate towards tumours and metastases in human breast tumour bearing mice [13].

It is generally accepted that 3D cell cultures are more similar to the composition of tumour microenvironment in vivo compared with 2D cell cultures [14–16]. Moreover, in vitro 3D cultures could fill the gap between 2D in vitro testing and in vivo animal models [17–18]. Spheroid-based 3D models are widely used to test cancer cell growth/proliferation, invasion, angiogenesis, and immune interactions and have been employed in drug screening and the development of new therapies [15–16]. Generally, a nonadherent surface coating (i.e., polyHEMA), hanging-drop assay or microfluidic devices are used to induce spheroid formation [16,19–21].

In the current study, we established a 3D co-culture model using QD-labelled MSCs to verify nanoparticle transfer between stromal and cancer cells in close spatial proximity. QDs are semiconductor nanocrystals with improved light emission, signal brightness, and resistance to photobleaching, thus making them a suitable imaging agent for the modelling of nanoparticle transfer [22].

The aim of the study was to demonstrate that nanoengineered MSCs can serve as a delivery vehicle to target breast cancer cells in a 3D co-culture model.

## Results

### Optimal quantum dot labelling conditions in mesenchymal stem cells

**Figure 1:**
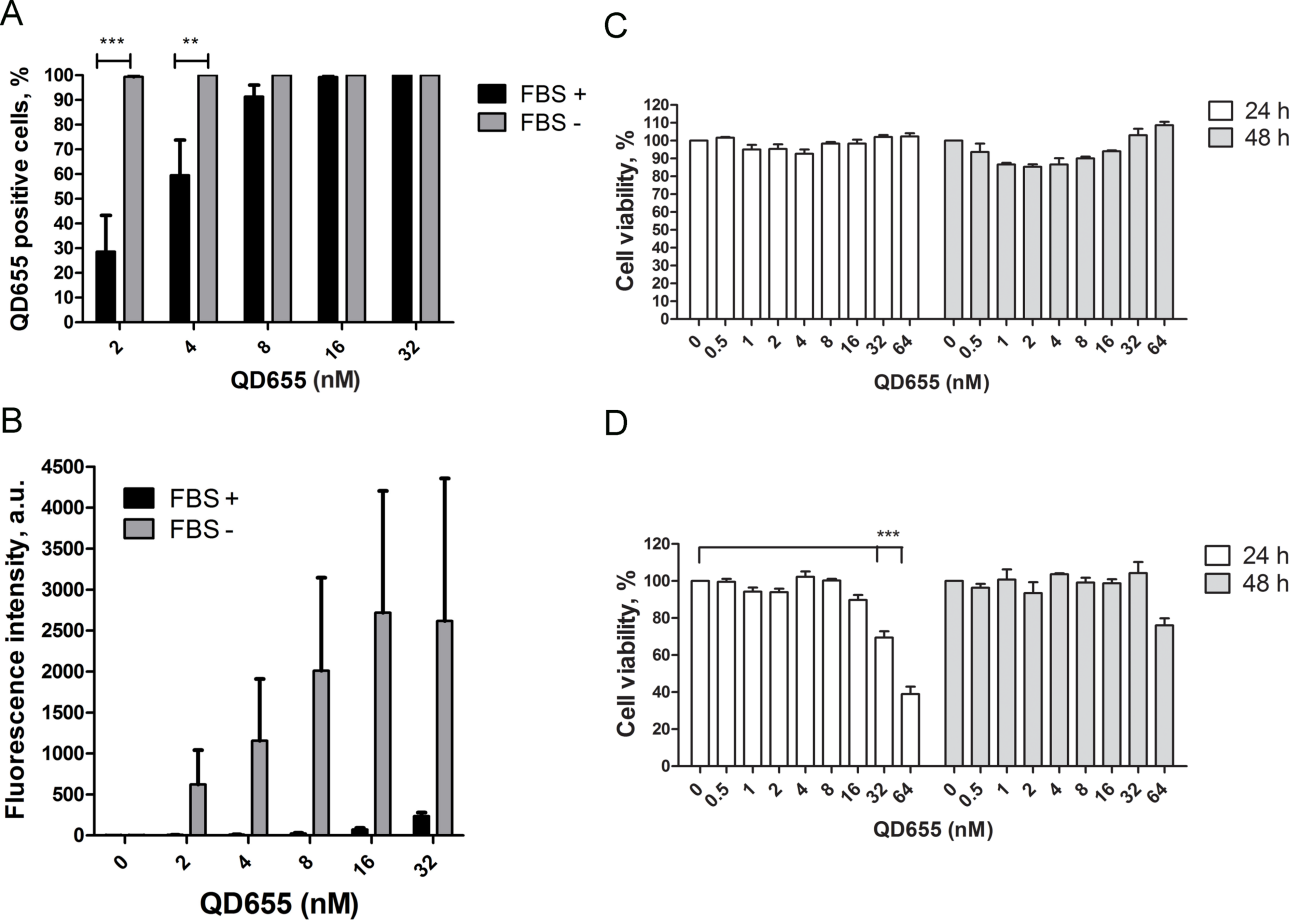
Characterisation of the optimal quantum dot (QD) incubation conditions in mesenchymal stem cells (MSCs). QD uptake after 6 h of incubation in MSCs cultivated in complete or serum-free medium expressed as the percentage of QD-positive cells (A) and the QD accumulation intensity (B). The impact of QDs on MSC viability after labelling in complete (C) and serum-free (D) medium following incubation for 24 h and 48 h. **p-value < 0.01, ***p-value < 0.001.

The flow cytometry data revealed that MSCs incubated in serum-free conditions accumulated more QDs compared with cells incubated in complete medium. At a QD concentration of 2 nM, 100% of the cells were labelled with QDs in serum-free medium, whereas a 100% positive cell population was achieved after incubation with 16 nM QDs in complete medium (Figure 1A). Fluorescence intensity analysis revealed that the QDs accumulated in the cells in a concentration-dependent manner. MSCs incubated with QDs diluted in the serum-free medium accumulated 100-fold more QDs compared with the complete medium (Figure 1B). Under serum-free conditions, QD uptake saturation was achieved at 16 nM, whereas no saturation was achieved in cells incubated with QDs in complete medium even at a concentration of 32 nM (Figure 1B).

Next, we analysed the MSC viability in response to intracellular QD accumulation. Incubation time points were selected at 24 h and 48 h to identify the QD-induced cytotoxic effects. We did not observe any cytotoxic effect on MSC viability when the QDs were applied in complete medium (Figure 1C). On the contrary, due to the 100-fold increase in the QD accumulation ratio under serum-free conditions, the QD toxicity was observed after 24 h of incubation with 32 nM and 64 nM QD by 30% and 50%, respectively (Figure 1D). Interestingly, the cytotoxic effect was not observed after 48 h of incubation with QDs, which could be explained by the reduction of intracellular QD concentration due to cell division. Thus, we chose a QD concentration of 16 nM as the optimal concentration for the labelling of cells in complete medium, whereas 8 nM was optimal for cell labelling in serum-free medium.

### Mesenchymal stem cell 3D culture model

**Figure 2:**
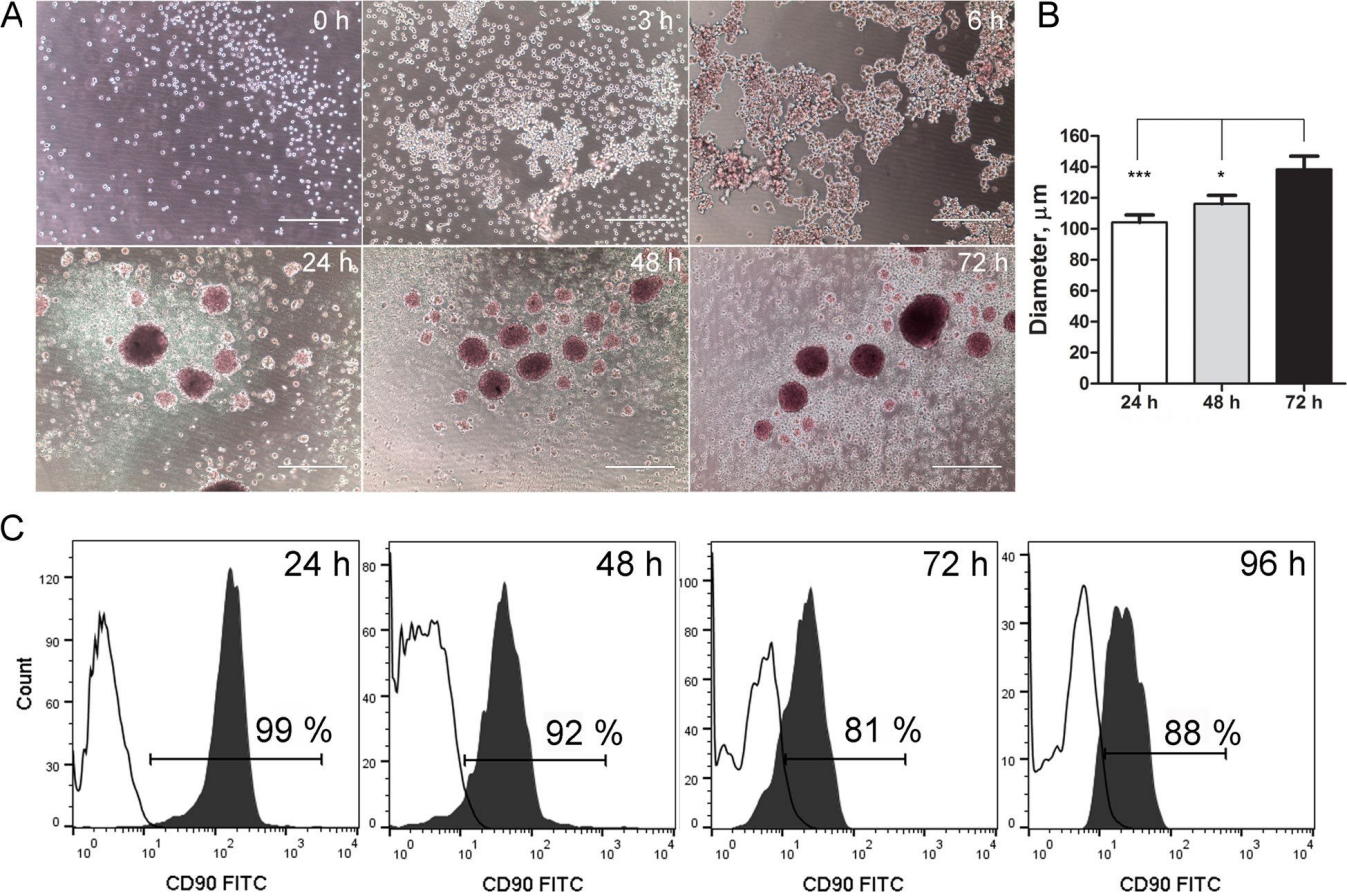
Characterisation of mesenchymal stem cells (MSCs) in 3D culture conditions. (A) MSC spheroid formation on polyHEMA coating. Scale bar – 400 μm. (B) The change in the diameter of MSC spheroids in 3D culture over time. (C) The dynamics of CD90 expression in 3D cultivated MSCs. *p-value < 0.05, ***p-value < 0.001.

MSC aggregation could be observed 3–6 h after seeding on poly(2-hydroxethyl methacrylate) (polyHEMA)-coated plates. 24 h later, the cells formed compact and dense floating spheroids of 100 μm in diameter (Figure 2A,B). The diameter of the spheroids further increased after 48 and 72 h (Figure 2A,B). Z-scan measurements revealed that live cells were present throughout the spheroid structure after 24 h of incubation (Supporting Information File 1, Figure S2).

CD90 was used as a selective marker for MSCs because it is not expressed in MCF7 and MDA-MB-231 cells [23]. To ensure the stability of the selective marker, the expression of CD90 in 3D MSC culture was monitored over time. After 24 h in spheroid culture, 97% of MSCs remained CD90 positive, which was similar to 2D culture [24]. However, after 48 h, 72 h and 96 h propagation on polyHEMA coatings, CD90 expression was reduced to 92%, 81% and 88%, respectively (Figure 2C). Therefore, we chose 24 h as the optimal incubation time for 3D cell co-culture experiments to ensure the selectivity of the CD90 marker towards MSCs.

### Quantum dot signal stability in mesenchymal stem cell spheroids

**Figure 3:**
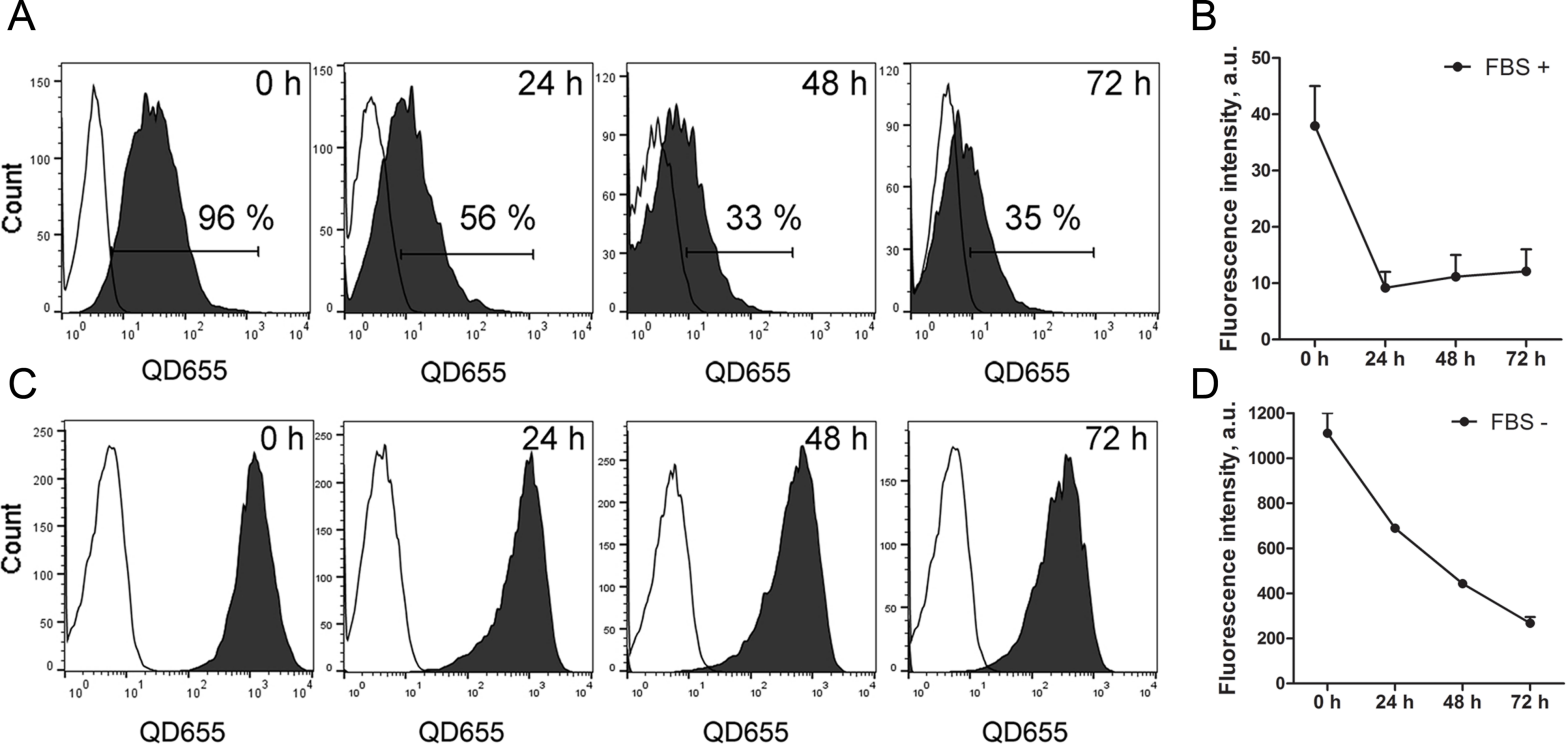
The dynamics of quantum dot (QD) signal in mesenchymal stem cells (MSCs) 3D culture. Representative data of MSC QD signal analysis in 3D culture after labelling in complete (A) or serum-free medium (C). The changes of the QD fluorescence signal intensity in the 3D MSC population after labelling in complete (B) and in serum-free (D) medium.

Next, we sought to determine the stability of the QD label in MSC spheroids. QD release was estimated in MSCs that were labelled with QDs in complete or serum-free medium in 2D culture and seeded on polyHEMA coatings to form spheroids. After 24 h, only 56% of MSCs that were labelled with QDs in complete medium and formed 3D structures had retained the QD signal. Following 48 h and 72 h of incubation, the number of QD-labelled MSCs in 3D culture decreased further to 33% and 35%, respectively (Figure 3A). The rapid reduction in the QD signal after 24 h was confirmed by fluorescence intensity analysis (Figure 3B). On the contrary, 100% of MSCs that were labelled in serum-free conditions remained QD positive until 72 h of incubation (Figure 3C). Despite the fact that 100% of the MSC population was QD positive in serum-free medium until 72 h, we observed a 5.5-fold/5-fold decrease in the fluorescence intensity, respectively, indicating that QD elimination occurs (Figure 3D). As mentioned previously, a significantly increased intracellular accumulation of QDs was observed in serum-free medium (Figure 1A,B), and the QD elimination effect was subsequently more pronounced (Figure 3D). Thus, we chose to label MSCs with QDs in serum-free medium to ensure the highest load of intracellular QDs for further 3D co-culture experiments.

### Quantum dot uptake in breast cancer cell 2D and 3D monocultures

**Figure 4:**
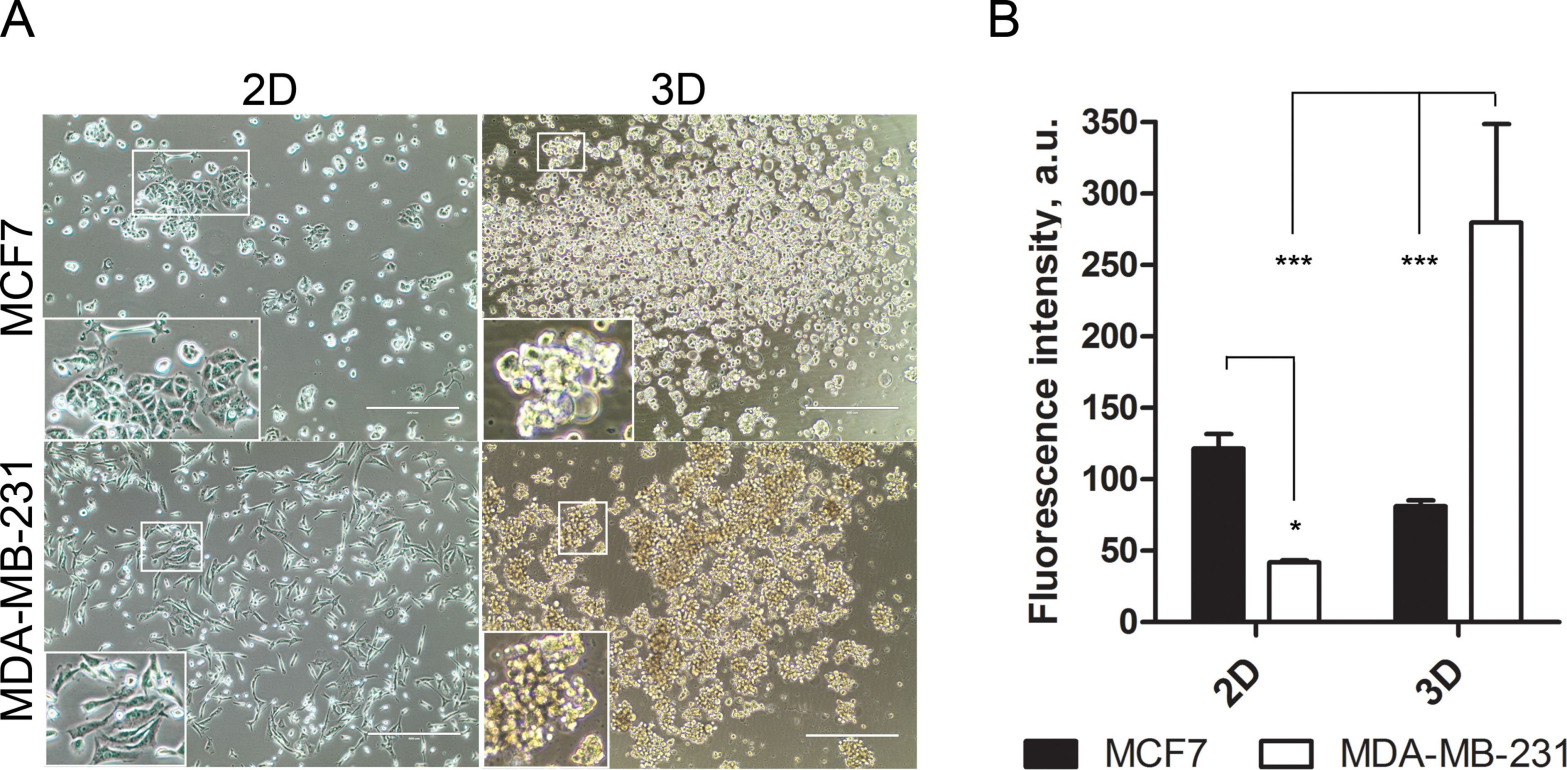
Comparison of MCF7 and MDA-MB-231 properties in 2D and 3D culture conditions. (A) The morphology of MCF7 and MDA-MB-231 cells in 2D and 3D culture. Scale bar – 400 μm. (B) Quantum dot (QD) uptake efficiency in MCF7 and MDA-MB-231 cells in 2D and 3D culture expressed as the intracellular QD fluorescence intensity. *p-value < 0.05, ***p-value < 0.001.

MCF7 and MDA-MB-231 cells formed loose, floating aggregates in 3D culture conditions (Figure 4A). MCF7 and MDA-MB-231 cells were labelled with 8 nM QDs in 2D and 3D culture to evaluate the differences in uptake efficiency under both conditions. We observed that MCF7 cells exhibited increased QD internalisation efficiency in standard culture conditions (2D) compared with 3D culture (Figure 4B). To the contrary, MDA-MB-231 internalised 6-fold more QDs in 3D culture compared with 2D (Figure 4B). Such discrepancy in uptake efficacy might be associated with different endocytosis pathways. MCF7 cells internalised QDs through phagocytosis and clathrin/caveolae-dependent endocytosis, whereas the clathrin/caveolae-dependent pathway dominated in MDA-MB-231 cells in monocultures (Supporting Information File 1, Figure S2).

### Cell viability in 3D culture

**Figure 5:**
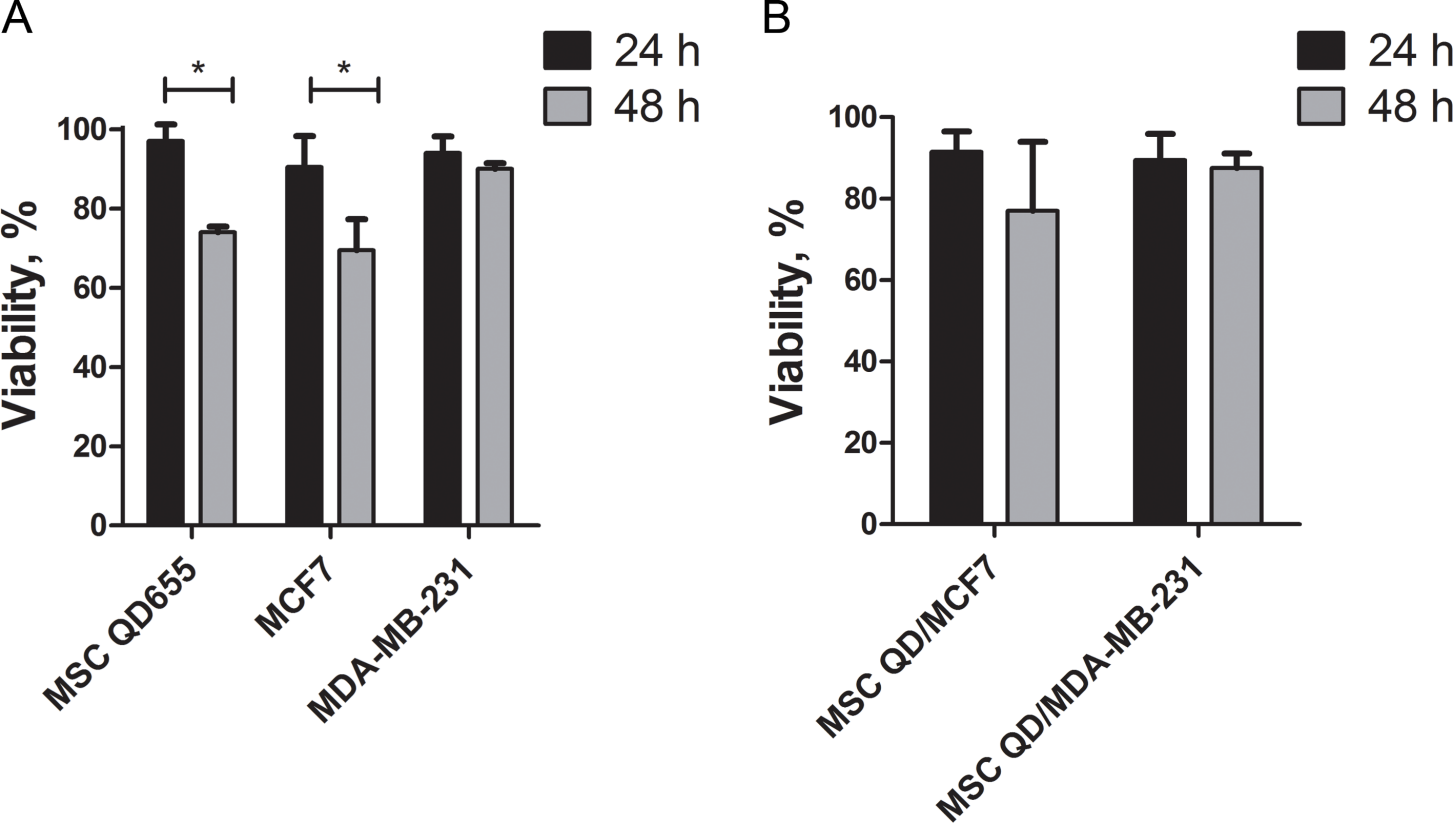
The viability of mesenchymal stem cells (MSCs), MCF7 and MDA-MB-231 cells in spheroids. The viability of cells was analysed in 3D monocultures (A) and 3D co-cultures (B) after 24 h and 48 h of cultivation on polyHEMA coatings. *p-value < 0.05.

Cells in a 3D culture formed floating and dense spheroids. Therefore, we sought to analyse the effect of the 3D culture conditions on cell viability (Figure 5). MSC and breast cancer cell populations were distinguished by CD90 expression, thus allowing viability estimations in each cell type separately. Cell viability in 2D culture was greater than 95% (data not shown). MSCs cultivated in 3D monocultures were fully viable after 24 h; nevertheless, a distinct decrease in viability of 26% was observed after 48 h (Figure 5A). MCF7 and MDA-MB-231 cell viability was not changed after 24 h. However, after 48 h, the viability of MCF7 cells was reduced by 31% (Figure 5A). The viability of MDA-MB-231 cells remained unchanged after 24 h and 48 h in 3D culture (Figure 5A). In 3D co-culture, MSC/MCF7 viability after 24 h decreased by 9%, of which 2% accounted for MSCs and 7% for MCF7. In MSC/MDA-MB-231 co-culture, 11% of cells were dead, of which 6% were MSCs and 5% were MDA-MB-231 after 24 h of cultivation. The cell survival rate in co-culture decreased after 48 h of propagation. The viability of cells in MSC/MCF7 co-culture decreased by 23% (10% MSCs and 13% MCF7), whereas the number of dead cells was 13% (6% MSCs and 7% MDA-MB-231) in MSC/MDA-MB-231 co-culture after 48 h. Viability was considered as another reason to select the 24 h incubation in 3D co-culture as the optimal time point for the study.

### Quantum dot transfer from mesenchymal stem cells to cancer cells in 3D co-culture

**Figure 6:**
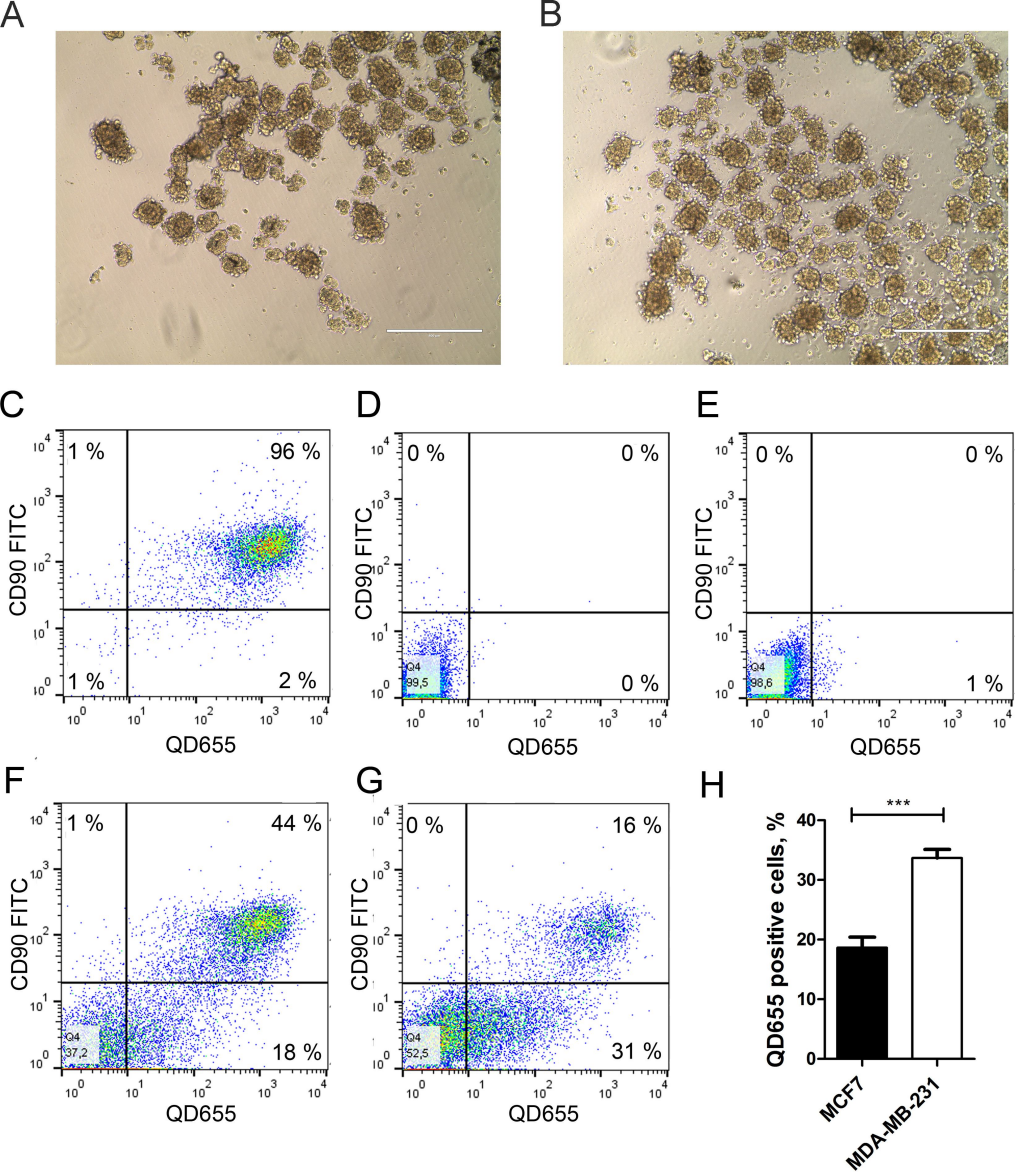
Quantum dot (QD) uptake in MCF7 and MDA-MB-231 cells in 3D co-culture. (A) Morphology of MSC/MCF7 and (B) MSC/MDA-MB-231 spheroids after 24 h of co-culture. Scale bar – 400 μm. (C) QD-loaded mesenchymal stem cells (MSCs), (D) MCF7 and (E) MDA-MB-231 cells were stained with CD90 to distinguish MSCs and cancer cell populations. Lower right quadrant in the dot plot shows the single QD positive population (QD-labelled cancer cells) in MSC/MCF7 (F) and MSC/MDA-MB-231 (G) 3D co-cultures. Representative data shown. (H) QD transfer efficiency from MSCs to cancer cells illustrated by the percentage of QD-positive/CD90-negative cells (*n* = 3). ***p-value < 0.001.

**Figure 7:**
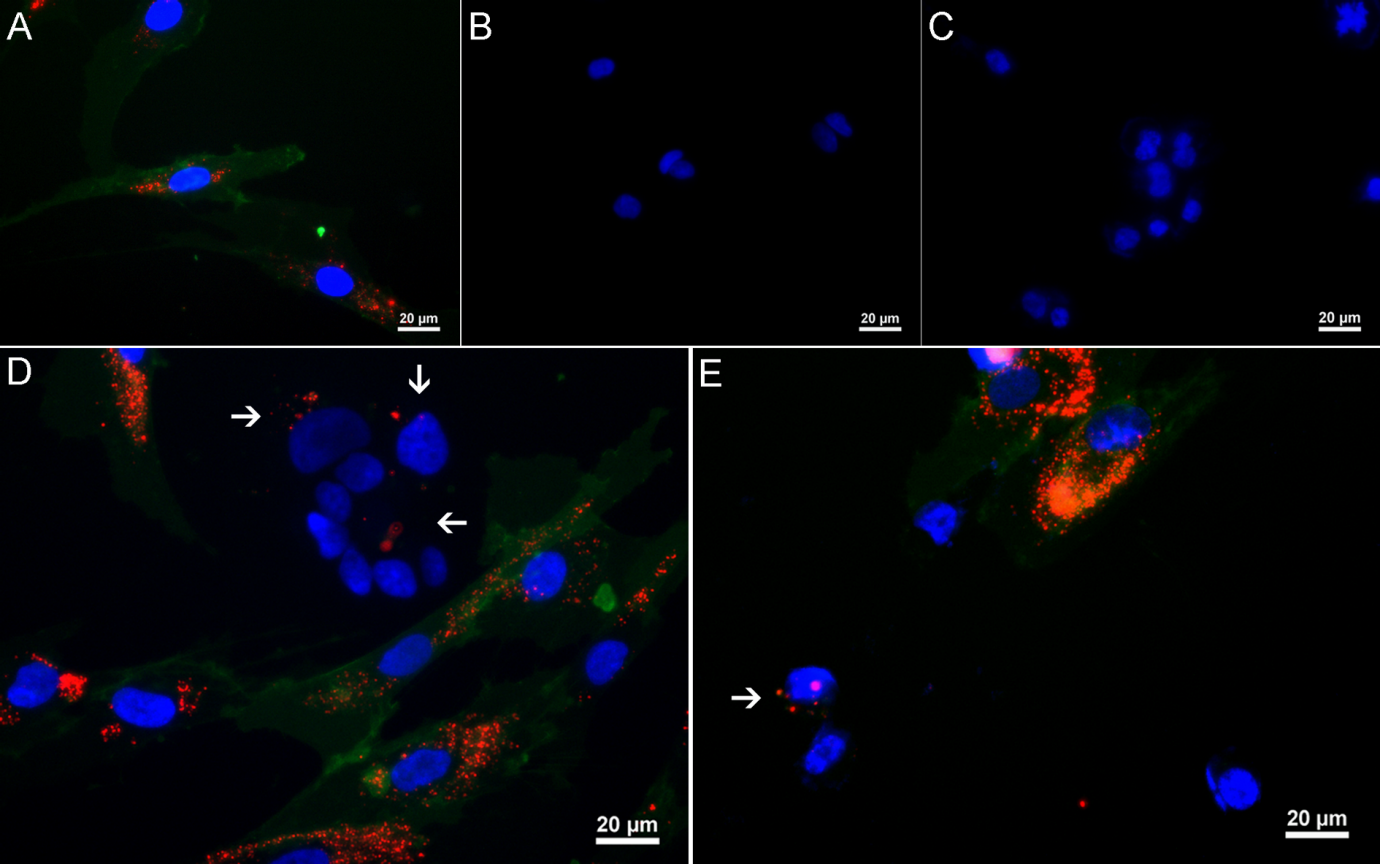
Fluorescence imaging of quantum dot (QD) intracellular accumulation in MCF7 and MDA-MB-231 cells during 3D co-culture. Nanoengineered MSCs (A), MCF7 (B) and MDA-MB-231 (C) cells were stained with CD90 FITC to distinguish cell populations. Single QD-positive/CD90-negative cells observed in MSC/MCF7 (D) and MSC/MDA-MB-231 (E) co-cultures. White arrows indicate cancer cells with internalised QDs. Blue – Hoechst, green – CD90 FITC, red – QDs. Scale bar – 20 μm.

The foremost aim of our study was to obtain experimental proof that nanoengineered MSCs could convey the QDs to the cancer cells in 3D co-culture conditions (Figure 6). Indeed, our data clearly demonstrate that after 24 h in 3D co-culture, 18% of MCF7 cells (Figure 6F) and 31% of MDA-MB-231 cells (Figure 6G) had internalised QDs as noted by the appearance of single QD label-positive cells in the lower right quadrant of the dot plot. Importantly, 96% of the QD-loaded MSC population was CD90 positive in 3D monoculture (Figure 6C). As expected, MCF7 (Figure 6D) and MDA-MB-231 cells (Figure 6E) were CD90 negative in 3D monocultures. The QD transfer from MSCs to cancer cells was also visualised by fluorescence imaging where CD90-negative/QD-positive cells represented cancer cells that have taken up the QDs released from MSCs during 3D co-culture (Figure 7). The proof of principle was additionally confirmed using cancer-cell-associated marker epithelial cell adhesion molecule (EpCAM) in nanoengineered MSC and MCF7 co-culture. Similarly to the previous data obtained with CD90 as a selective marker in the co-culture model, the QD transfer efficiency from nanoengineered MSCs to MCF7 cells was on average 18% (see Supporting Information File 1, Figure S3).

## Discussion

MSCs and the cancer cell 3D co-culture model described in this study simulate tumour microenvironment conditions in vivo and allow the evaluation of nanoparticle transfer between different cell types. The most important finding in the current 3D co-culture model is that intercellular QD delivery occurs between nanoengineered MSCs and breast cancer cells. The transcellular crosstalk between stromal cells and breast cancer cells has been studied previously in MSC/MDA-MB-231 co-cultures using the hanging drop method [25]. Pietila et al. demonstrated that cell–cell interaction is required for mortalin-conjugated QD655 transfer from MSCs to cancer cells, whereas no QD transfer occurred in a trans-well 2D system lacking cell contact [25]. The direct intercellular transfer of mortalin–QD655 between MSCs and breast cancer cells occurred through the formation of nanotubes or cell–cell fusion [26–28]. Similarly, it has been previously reported that QDs are actively transported between cardiac myocytes using membrane nanotubes [29]. In our study, we demonstrate that MSCs efficiently uptake QDs in serum-free conditions and then excrete the QDs, which then accumulate in MDA-MB-231 and MCF7 breast cancer cell lines in the 3D co-culture (Figure 3C,D). Noteworthy is that the permeability glycoprotein (P-glycoprotein)-mediated excretion of QDs from stem cells has been reported in other studies [30–32]. In our co-culture model, QDs are likely lost from MSCs via P-glycoprotein excretion. In 3D co-culture, MSCs and cancer cells are in close spatial proximity (Figure 6A,B), thus facilitating the uptake of MSC-excreted QDs by cancer cells. Our data demonstrate that phagocytosis and clathrin/caveolae-dependent endocytosis are the major QD uptake pathways in MCF7 cells, whereas the clathrin/caveolae-dependent pathway dominated in MDA-MB-231 cells in monocultures (see Supporting Information File 1, Figure S1). These findings are consistent with previous studies that demonstrated clathrin-dependent endocytosis, micropinocytosis and caveolae-mediated endocytosis as the main routes for NP uptake in MCF7 and MDA-MB-231 cells [33–35]. Nevertheless, we were not able to analyse the uptake pathway in 3D conditions likely due to poor inhibitor penetrance into the spheroids.

In general, 3D cultures are extensively studied given their potential to mimic cellular interactions and signal transduction occurring in in vivo conditions [14]. Briefly, 3D cell culture formation occurs on natural compounds, such as chitosan and hyaluronic acid, or synthetic agents, such as polyHEMA and poly(vinyl alcohol) (PVA) [14,36]. PolyHEMA has been widely used to induce spheroid formation in cancer cells and cells from healthy tissue [37–38]. Notably, the cell morphology and even phenotype changes in 3D culture comparing to the conventional 2D culture. We observed a decrease in the expression of CD90 following 48 h propagation in 3D conditions, which could be explained by a cell reaction to the change of the microenvironment or cell differentiation [39–40]. Similar effects were observed in bone marrow MSCs where CD29, CD44, CD73, CD90, and CD105 expression decreased after 7 days in 3D culture. To the contrary, haematopoietic marker CD34 and CD45 expression was increased [41]. CD90 is one of the key markers used for MSC characterisation [24]. In our hands Hoechst, 3,3’-dioctadecyloxacarbocyanine perchlorate (DiO), calcein, PKH67 and baculovirus gene transfer into mammalian cells (BacMam) transfection lacked cell type- selective specificity (data not shown). Thus, we used CD90 as a selective marker for MSCs because it is not expressed on MCF7 and MDA-MB-231 cells [23]. Additionally, we used EpCAM as a selective marker for breast cancer cells. It has been reported that the MSC marker expression profile can be changed in a co-culture with cancer cells and vice versa [28,42]. Slight CD90 expression can be induced in cancer cells after 48–72 h [28]. Similarly, cancer cell markers such as EpCAM can be induced in MSCs after 72 h of co-culture with breast cancer cells [42]. However, such marker induction has not been reported after 24 h of co-culture. Therefore, 24 h serve as a suitable co-culture time to analyse the QD transfer from MSCs to cancer cells. EpCAM expression was detected only in MCF7 cells and not in MDA-MB-231 cells, therefore the proof of principle of QD transfer from nanoengineered MSCs to breast cancer cells was demonstrated using MCF7 cells only (Supporting Information File 1, Figures S3 and S4).

Cell viability in spheroids is another critical issue that could affect the outcome of the co-culture experiment. In our study, we did not observe the formation of necrotic cells after 24 h (Supporting Information File 1, Figure S2), and cell viability was not affected (Figure 5). The size of the spheroids increased each day; therefore, a slight decrease in viability began after 48 h (Figure 5). The ability of the cells to survive in a 3D environment might be cell source dependent. For example, umbilical cord MSCs did not form necrotic zones up to 11 days in the 3D culture [43].

Metastatic cancer cells are resistant to extracellular matrix detachment-induced apoptosis (anoikis) [44]. Anoikis resistance therefore determines the cell survival and behaviour in a 3D culture. It was previously shown that MDA-MB-231 cells in 3D culture remains viable up to 48 h; however, remarkable MCF7 cell death occurs after 24 h in a 3D culture [45]. Despite the general assumption that the uptake processes of nutrients and NPs are reduced in 3D culture, our data indicate that QD accumulation is increased in MDA-MB-231 cells in 3D culture conditions compared with 2D (Figure 4B). The same tendency was also confirmed in our 3D co-culture study in which MDA-MB-231 cells internalised almost twice as much QDs compared with MCF7 cells (Figure 6F–H). Thus, we demonstrated that nanoengineered MSCs could be a tool for targeting metastatic breast cancer cells. It has been reported that metastatic breast cancer cells demonstrate higher nanocarrier uptake efficiency than MCF7 due to the overexpression of integrin receptors which mediate the uptake of NPs through integrin receptor mediated endocytosis [46].

Ex vivo injected MSCs have a relatively short lifespan within the body. On average, 24 h after the injection, these MSCs are relocated to the liver and spleen [47–48]. MSCs overexpress the drug efflux transporter P-glycoprotein, which ensures rapid excretion of toxic substances from MSCs. Thus, MSCs are an excellent vector for low cytotoxicity anticancer drugs [4]. Sadhukha et al. demonstrated that nanoengineered MSCs home to A549 lung cancer in vivo and remain there for at least 3 h, which could be sufficient time to release drugs into the tumour. The encapsulation of NP-linked anticancer drugs could ensure metered drug release in tumours [2]. QD linkage to photosensitisers, such as chlorin e_6_, causes damage to MiaPaCa2 cancer cells via light-induced cytotoxicity, demonstrating a promising approach for NP-based cancer therapies [49]. These approaches indicate that MSCs could be used as potential drug delivery vectors in tumour therapies.

## Conclusion

In summary, we demonstrated the feasibility of a 3D co-culture model to study targeted drug delivery by nanoengineered MSCs. For NP delivery purposes, MSC labelling with QDs in serum-free medium ensures increased loading efficiency. The QD transfer from MSCs is more efficient in co-culture with the metastatic breast cancer cell line MDA-MB-231 compared with non-metastatic MCF7 cells. Thus, nanoengineered MSCs could be considered as nanoparticle delivery vehicles to specifically target metastatic breast cancer cells.

## Experimental

### Cell culture

Primary human skin mesenchymal stem cells (MSCs) from frozen primary cell stock were used in accordance with authorised approval from the Institute of Experimental and Clinical Medicine Ethics Committee, University of Latvia (issued 04.06.2014) as described previously [24]. The cells were propagated in the medium Dulbecco’s modified Eagle’s medium/nutrient mixture F-12 (DMEM/F12) (3:1 v/v) supplemented with 10% of fetal bovine serum (FBS) and antibiotics (100 U/mL penicillin, 100 μg/mL streptomycin) (complete MSC medium). Cell suspensions were propagated in tissue culture flasks until 80% confluence in a humidified chamber at 37 °C with 5% CO_2_. MSCs from passages 3 to 6 were used in experiments. The cells cultivated in complete or serum-free medium (DMEM/F12 3:1) were used for the experiments.

Human breast cancer cell lines MDA-MB-231 (ATCC HTB-26™) and MCF7 (ATCC HTB-22™) were propagated in DMEM supplemented with 10% FBS and penicillin/streptomycin (100 U/mL and 100 μg/mL, respectively) (complete cancer cell medium). The cells were cultured in 25 cm^2^ polystyrene tissue culture flasks up to 90% confluence in complete cell culture medium in a humidified chamber at 37 °C with 5% CO_2_.

For passaging, the cells were trypsinised using 0.25% trypsin–EDTA solution. All cell reagents were purchased from Sigma-Aldrich, St. Louis, MO, USA.

### Establishment of 3D cell culture model

A poly(2-hydroxethyl methacrylate) (polyHEMA) coating was prepared as described elsewhere [50]. In brief, the PolyHEMA solution was poured into the wells of a 24-well tissue culture polystyrene plate to cover the surface. The plate was then air dried in a laminar airflow chamber overnight. The MSCs, MDA-MB-231 and MCF7 cells were seeded at a density 5 × 10^4^ cells per well on polyHEMA-coated plates in the complete cell culture media. Then, the 3D spheroid formation was analysed using an EVOS XL light transmission microscope at 24, 48 and 72 h (AMG, Washington, USA).

To distinguish between cell populations in the co-culture, CD90 was chosen as a selective marker for MSCs and EpCAM was chosen as a marker for MCF7 cells. CD90 expression dynamics were analysed in MSCs after 24, 48, 72 and 96 h of propagation in 3D culture. The spheroids were pelleted by centrifugation at 250 g for 5 min, trypsinised for 5 min at 37 °C to obtain a single cell suspension, and finally centrifuged and suspended in 100 μL of PBS. The samples were stained with FITC mouse anti-human CD90 (clone 5E10, BD Bioscience, Carlsbad, CA, USA) or FITC mouse anti-human EpCAM (clone EBA-1, BD Bioscience, Carlsbad, CA, USA) for 30 min at room temperature. Flow cytometry data were acquired on a Guava EasyCyte 8HT flow cytometer and analysed by ExpressPro software (Millipore, MA, USA).

### Quantum dots

Qdot® 655 ITK™ non-targeted carboxyl-coated quantum dots were purchased from Thermo Fisher Scientific, USA. The QDs are composed of a CdSe core and ZnS shell coated with an amphiphilic polymer and functionalised with carboxylate. The QDs have an emission maxima of 655 nm. Xu et al. reported that the hydrodynamic diameter of the nanoparticles is 14.55 ± 4.157 nm and the zeta potential is −35.1 mV [51]. The stock solution was prepared at a concentration of 8 µM in 50 mM borate with pH 9.0. Further preparations of the QD solution are described at each methodological part separately.

### Preparation of nanoengineered cells

Carboxyl QD655 (Thermo Fisher Scientific, Waltham, Massachusetts, USA) was used in the study. To estimate the optimal QD concentration for uptake experiments, 5 × 10^4^ MSCs were allowed to adhere to 6-well tissue culture polystyrene (TCPS) plates and cultured in the presence of QDs of 2 nM to 32 nM concentration for 6 h in complete or serum-free medium. The cells were then harvested by trypsinisation and resuspended in 200 μL PBS for further studies.

### Cell viability assay

The effect of QDs on MSC viability was analysed using the cell counting kit 8 (CCK8) (Sigma-Aldrich, St. Louis, MO, USA). Briefly, 5 × 10^3^ cells per well were seeded on a 96-well plate in 100 μL of complete medium. On the next day, QDs at concentrations ranging from 0.5 to 64 nM were added in serial dilutions using a 2-fold dilution factor either in complete or serum-free medium. The cells in complete medium were incubated with QDs for 24 and 48 h. The cells in serum-free medium were incubated with QDs for 6 h followed by replenishment with fresh complete medium. The cells were subsequently incubated for 24 and 48 h. After incubation, 10 µL of CCK8 reagent was added to each well, and the cells were incubated for 2 h at 37 °C in 5% CO_2_ and 90% humidity. The background signal controls containing QDs at all tested concentrations in the cell culture medium were introduced. The optical density was measured using a BioTek ELx808 spectrophotometer (BioTek Instruments, Winooski, VT, USA) at a wavelength of 450 nm.

### Quantum dot release assay

To analyse the release of the QDs from the MSCs in the 3D culture, the cells were labelled with 8 nM QDs in serum-free medium or 16 nM QDs in complete medium for 6 h on TCPS plates. The cells were then trypsinised and seeded in polyHEMA-coated plates in complete medium. MSC spheroids were harvested at 24, 48 and 72 h and trypsinised for 5 min at 37 °C to obtain a single cell suspension. The QD signal was analysed by flow cytometry as described in the section “Establishment of 3D cell culture model”.

### 3D co-culture model for quantum dot transfer analysis

Prior to 3D co-culture seeding, 1 × 10^5^ MSCs were labelled with 8 nM QDs for 6 h in serum-free medium. The cells were then trypsinised, and 5 × 10^4^ MSCs were seeded in polyHEMA-coated plates for co-culture with 2.5 × 10^4^ MCF7 or MDA-MB-231 cells (2:1) in complete medium [52–53]. After 24 h, supernatants containing floating spheroids were aspirated and centrifuged at 250 g for 5 min. The pellet was suspended in 0.25% trypsin–EDTA for 5 min at 37 °C to ensure the dissociation of the spheroids into a single cell suspension. The cells were washed, stained with CD90 FITC or EpCAM FITC and analysed by flow cytometry.

For fluorescence imaging of QD transfer, the cancer cell and MSC mono- or co-culture spheroids were harvested after 24 h of propagation on the polyHEMA coating, trypsinised to obtain single cell suspension and then allowed to adhere to microscopy chamber slides overnight in complete medium. Next, the cells were stained with human CD90 FITC (clone DG3, Miltenyi Biotec, Bergisch Gladbach, Germany) diluted 1:11 in complete medium for 1 h at room temperature. The samples were then counterstained with Hoechst 33342 trihydrochloride (10 mg/mL) solution (Thermo Fisher Scientific, USA), fixed with 4% PFA and mounted with ProLong gold anti-fade mounting medium.

### Fluorescence imaging

For the z-scan measurements, MSC spheroids were stained with 5 μM 3,3’-dioctadecyloxacarbocyanine perchlorate (DiO) in complete medium for 45 min at 37 °C and counterstained with Hoechst dye. The cells were then trypsinised and seeded in 3D culture for 24 h. The spheroids were then transferred to 4-well chamber slides. All reagents were obtained from Thermo Fisher Scientific, Waltham, Massachusetts, USA.

Fluorescence imaging was performed as described previously [54]. A Nikon eclipse Ti microscope equipped with a Nikon C2 confocal system was used. A Nikon S Plan Fluor ELWD 40×/0.60 objective was used. A 488 nm laser was used to excite CD90 FITC and DiO. In addition, a 405 nm laser was used to excite Hoechst and QD655. To detect the fluorescence, the following filters were used: 447/60 nm with Hoechst 525/50 nm for CD90 FITC and DiO, and a 561 long pass filter for QD655 (all from Nikon, Tokyo, Japan). Each channel was recorded separately to avoid spectral overlap. The images were analysed using Nis-Elements C 4.13 software (Nikon, Tokyo, Japan). Quantification of the QD fluorescence signal was performed using Nis-Elements C 4.13 software.

### Cell viability in 3D culture

Cell viability in the 3D culture was analysed by propidium iodide (PI, Sigma-Aldrich, St. Louis, MO, USA). MSCs, MCF7 and MDA-MB-231 mono- or co-culture spheroids were harvested from 3D culture after 24 and 48 h, trypsinised and stained with FITC mouse anti-human CD90 (clone 5E10). The samples were then treated with 10 μg/mL PI at a 1:40 dilution in PBS for 5 min at room temperature and analysed by flow cytometry.

### Statistical analysis

Statistical analysis was performed using GraphPad Prism Software (GraphPad Inc., California, USA). Data were expressed as the mean ± standard error of mean. The differences between the studied groups (*n* = 3) were statistically assessed by one-way ANOVA followed by Tukey’s post hoc test. The significance was represented as follows: *p-value < 0.05, **p-value < 0.01, ***p-value < 0.001.

## Supporting Information

File 1 Additional experimental information.
Endocytosis inhibitor assay; QD endocytic pathway analysis in MSCs, MCF7 and MDA-MB-231 cells; Confocal microscopy z-sections of MSC and breast cancer cell co-culture spheroids; EpCAM expression in MSCs and breast cancer cells MCF7 and MDA-MB-231; QD transfer from nanoengineered MSCs to MCF7 cells using EpCAM as a selective marker for MCF7 cells.
