# Supplementary material for: Nanoparticle delivery to metastatic breast cancer cells by nanoengineered mesenchymal stem cells
Source: Beilstein J Nanotechnol. 2018 Jan 29;9:321–32. doi: 10.3762/bjnano.9.32 (PMC5815277; doi:10.3762/bjnano.9.32)
Supplement: File 1 — Additional experimental information. Endocytosis inhibitor assay; QD endocytic pathway analysis in MSCs, MCF7 and MDA-MB-231 cells; Confocal microscopy z-sections of MSC and breast cancer cell co-culture spheroids; EpCAM expression in MSCs and breast cancer cells MCF7 and MDA-MB-231; QD transfer from nanoengineered MSCs to MCF7 cells using EpCAM as a selective marker for MCF7 cells. [file Beilstein_J_Nanotechnol-09-321-s001.pdf]

Supporting Information

for

Nanoparticle delivery to metastatic breast cancer cells by nanoengineered mesenchymal stem cells

Liga Saulite[1*], Karlis Pleiko[1], Ineta Popena[1], Dominyka Dapkute[2,3], Ricardas Rotomskis[2,4], and Una Riekstina[1]

Address: [1]Faculty of Medicine, University of Latvia, Raina Blvd. 19, LV-1586 Riga, Latvia, [2]Biomedical Physics Laboratory, National Cancer Institute, P. Baublio Street 3b, LT-08406 Vilnius, Lithuania, [3]Life Science Center, Vilnius University, Sauletekio Ave. 7, LT-10257 Vilnius, Lithuania, and [4]Laser Research Centre, Vilnius University, Sauletekio al. 9, corp. 3, LT-10222 Vilnius, Lithuania

* Corresponding author

Email: Liga Saulite – liga.saulite@lu.lv

Additional experimental information

Endocytosis inhibitor assay – method description

To analyse the pathway of QD uptake in MSCs, MCF7 and MDA-MB-231, five endocytosis inhibitors were selected: clathrin pathway inhibitor chlorpromazine (CPZ), phagocytosis inhibitor cytochalasin D (CytD), macropinocytosis inhibitor ethylisopropylamiloride (EIPA, Cayman Chemical, Michigan, USA), lipid raft inhibitor nystatin and clathrin/caveolae-dependent pathway inhibitor dynasore (all from Sigma-Aldrich, St. Louis, MO, USA if not stated otherwise). MSC, MCF7 and MDA-MB-231 cells were pre-treated for 1 h with inhibitors at the following concentrations: 40 µM CPZ, 2 µM CytD, 5 µM EIPA, 80 µM nystatin and 80 µM dynasore at 37 °C, 5 % CO_2 and 95 % humidity. Medium was then aspirated. MSCs were incubated with 16 nM QDs in complete or 8 nM QDs in serum-free medium for 6 h. MCF7 and MDA-MB-231 cells were incubated with 8 nM QDs for 1 h in complete medium. Medium was aspirated, and samples were rinsed with PBS thrice. Control wells contained untreated cells. Cells were fixed by 4 % PFA, permeabilised with 0.3 % Triton X-100 (all from Sigma-Aldrich, St. Louis, MO, USA) and stained with methanolic Alexa Fluor 488 Phalloidin (1:100) and Hoechst (1:1000). Samples were analysed by immunofluorescent staining.

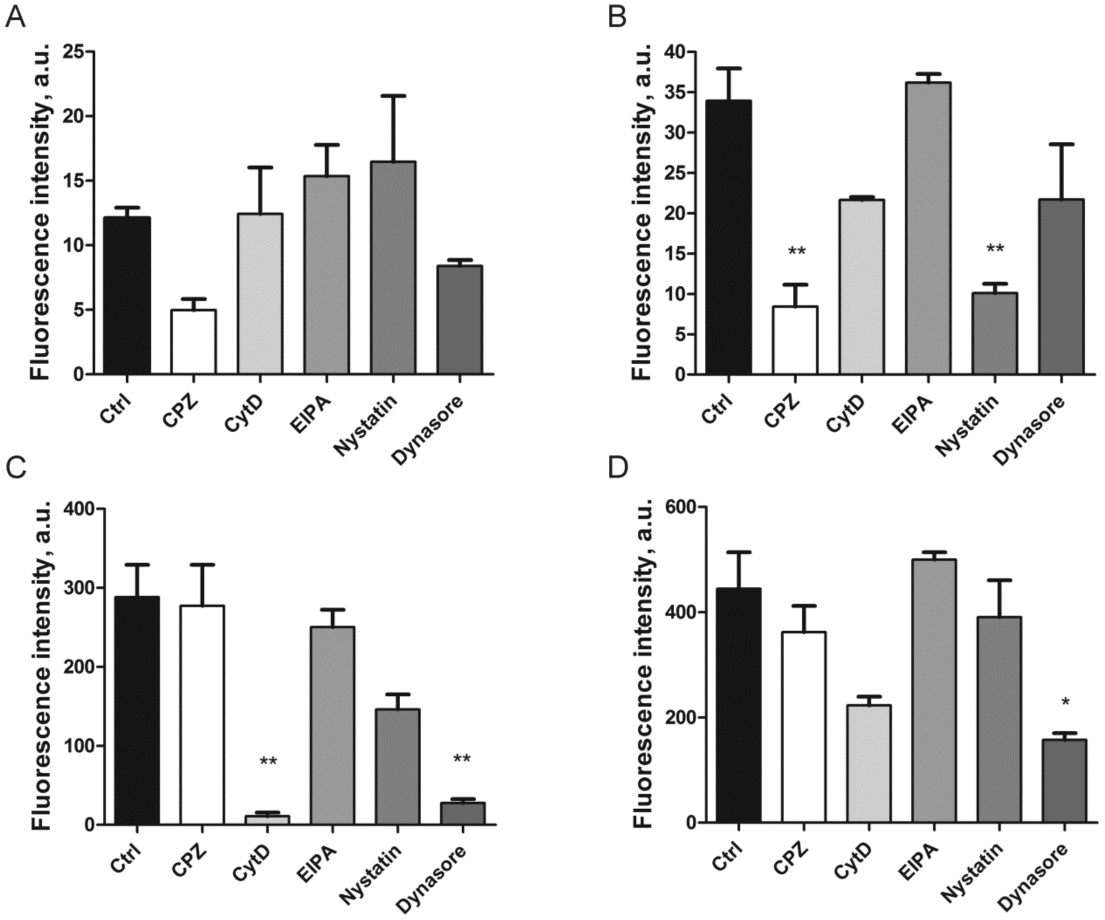

Figure S1: Results showing the main endocytic pathways involved in the uptake of QDs in MSCs cultivated in (A) serum containing and (B) serum-free medium, (C) MCF7 and (D) MDA-MB-231 cells.

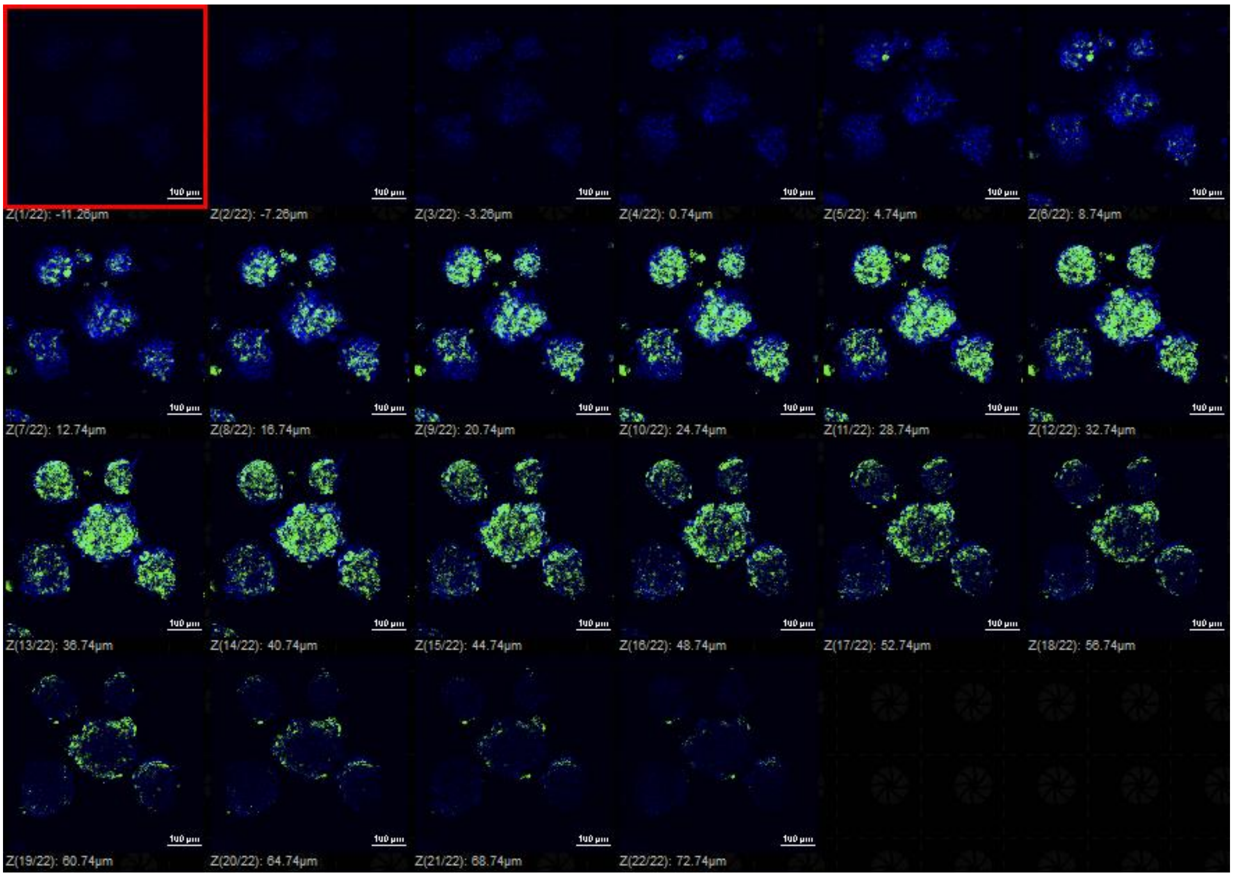

Figure S2: Z-stack confocal microscopy analysis of MSC/breast cancer cell co-culture spheroids.

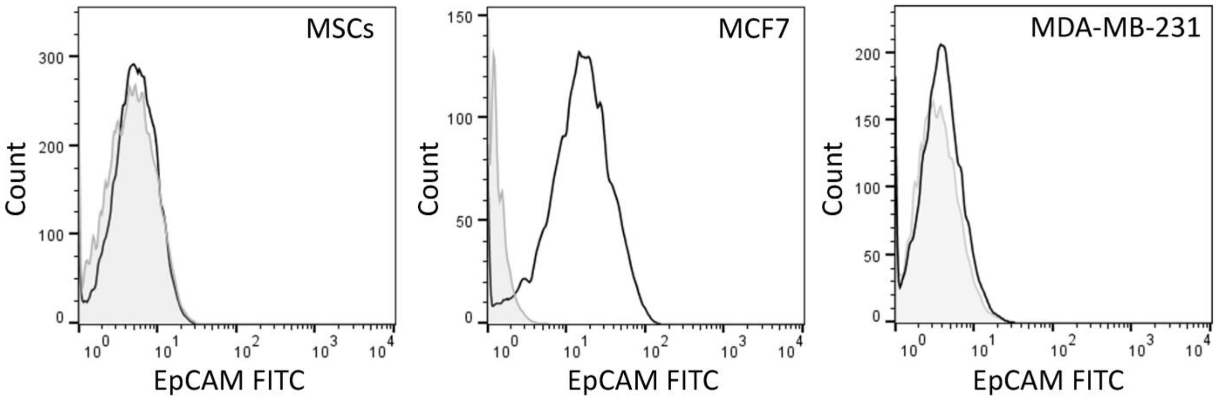

Figure S3: Cancer marker EpCAM expression was tested in MSCs, primary breast cancer cells MCF7 and metastatic breast cancer cells MDA-MB-231. EpCAM expression was observed only in MCF7 cells. Open histogram – EpCAM stained cells, grey histogram – negative control.

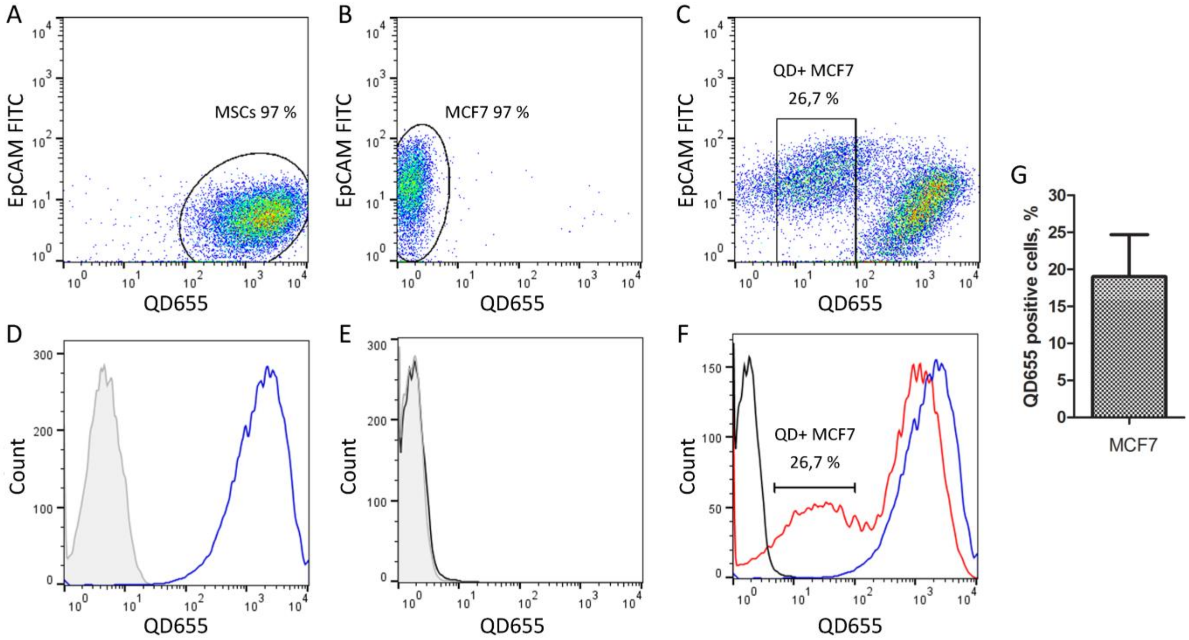

Figure S4: QD transfer from nano-engineered MSCs to MCF7 cells using EpCAM as a selective marker for MCF7 cells. Co-culture populations were separated by marker expression and QD fluorescence. (A, D) nanoengineered MSCs labelled with QDs and EpCAM antibody (blue histogram), (B, E) MCF7 cells stained with EpCAM antibody (black histogram), (C, F) nano-engineered MSC and EpCAM antibody stained MCF7 co-culture (red histogram) (representative sample shown). (G) QD transfer efficiency from nanoengineered MSCs to MCF7 cells after 24 h of co-culture 3D (n = 3). On average 18% of MCF7 cells uptake QDs from nanoengineered MSCs (n = 3).
